# MP470, a novel receptor tyrosine kinase inhibitor, in combination with Erlotinib inhibits the HER family/PI3K/Akt pathway and tumor growth in prostate cancer

**DOI:** 10.1186/1471-2407-9-142

**Published:** 2009-05-11

**Authors:** Wenqing Qi, Larry S Cooke, Amy Stejskal, Christopher Riley, Kimiko Della Croce, Jose W Saldanha, David Bearss, Daruka Mahadevan

**Affiliations:** 1 Arizona Cancer Center, the University of Arizona, Tucson, AZ 85724, USA; 2 National Institute for Medical Research, Mill Hill, London, UK; 3 SuperGen, Inc, Dublin, CA 94568, USA

## Abstract

**Background:**

Prostate cancer is a common disease in men and at present there is no effective therapy available due to its recurrence despite androgen deprivation therapy. The epidermal growth factor receptor family (EGFR/HER1, HER2/neu and HER3)/PI3K/Akt signaling axis has been implicated in prostate cancer development and progression. However, Erlotinib, an EGFR tyrosine kinase inhibitor, has less effect on proliferation and apoptosis in prostate cancer cell lines. In this study, we evaluate whether MP470, a novel receptor tyrosine kinase inhibitor alone or in combination with Erlotinib has inhibitory effect on prostate cancer *in vitro *and *in vivo*.

**Methods:**

The efficacy of MP470 or MP470 plus Erlotinib was evaluated *in vitro *using three prostate cancer cell lines by MTS and apoptosis assays. The molecular mechanism study was carried out by phosphorylation antibody array, immunoblotting and immunohistochemistry. A LNCaP mouse xenograft model was also used to determine the tumor growth inhibition by MP470, Erlotinib or the combination treatments.

**Results:**

MP470 exhibits low μM IC_50 _in prostate cancer cell lines. Additive effects on both cytotoxicity and induction of apoptosis were observed when LNCaP were treated with MP470 in combination with Erlotinib. This combination treatment completely inhibited phosphorylation of the HER family members (HER1, 2, 3), binding of PI3K regulatory unit p85 to HER3 and downstream Akt activity even after androgen depletion. Furthermore, in a LNCaP mouse xenograft model, the MP470-Erlotinib combination produced 30–65% dose-dependent tumor growth inhibition (TGI).

**Conclusion:**

We propose that MP470-Erlotinib targets the HER family/PI3K/Akt pathway and may represent a novel therapeutic strategy for prostate cancer.

## Background

Prostate cancer is one of the leading causes of cancer mortality in men, with an estimated 218,890 new patients and 27,050 deaths in the US in 2007 [1]. Utilization of prostate-specific antigen (PSA) as a surrogate biomarker results in earlier diagnosis of the disease [2]. Localized disease can be cured with radical prostatectomy or radiotherapy [3]. However, patients with advanced or bulky local disease are at increased risk of treatment failure following local therapy [4]. Most patients remain largely asymptomatic until the development of overt metastatic disease. The current gold standard in men with newly diagnosed metastatic disease is androgen deprivation therapy (ADT) [5] which decreases the volume of the primary and metastatic lesions by inducing apoptosis [6]. In most cases, after an initial response, tumors recur as hormone-refractory prostate cancer (HRPC) and are unresponsive to additional androgen withdrawal [7]. Clinical trials of taxane-based therapy in HRPC have demonstrated a survival benefit and increased time to progression [8]. However, this therapy is not curative. Clinical trials are evaluating novel regimens, including platinum agents (satraplatin), microtubule stabilizing agents (epothilone B), mammalian target of rapamycin (everolimus) and immunotherapeutic vaccines [9]. Despite these advances, novel effective therapies for prostate cancer based on mechanism of action studies are urgently needed.

Receptor tyrosine kinases (RTKs) have emerged as new drugable targets for treatment of several human solid and hematological malignancies [10,11]. For example, imatinib mesylate (IM, Gleevec; Novartis), an inhibitor of Bcr-Abl, c-Kit and platelet-derived growth factor receptor (PDGFR), has been successfully used in the treatments of chronic myeloid leukemia (CML) and gastrointestinal stromal tumors (GISTs) [12]. Erlotinib (Tarceva; OSI Pharmaceuticals), an inhibitor of the epidermal growth factor receptor (EGFR), is also approved for the treatment of patients with locally advanced or metastatic non-small cell lung cancer and pancreatic carcinoma in combination with gemcitabine [13]. RTKs are trans-membrane proteins with a ligand-binding extracellular domain and a catalytic intracellular kinase domain. The enzymatic activity of RTKs is under tight control, so that non-proliferating cells have very low levels of tyrosyl phosphorylated proteins. Ligand binding leads to activation of the RTK and subsequent downstream signaling through the PI3K/Akt pathway [14,15].

In human prostate cancer several RTKs including the EGFR family (HER1, 2, and 3), PDGFR (alpha, beta), c-Ret and ephrin (EPH) are over-expressed compared to normal prostatic tissue [16-18], implicating pivotal roles in tumorigenesis. Importantly, their downstream signaling leads to constitutive activation of the PI3K/Akt pathway [19,20], an important intracellular mediator involved in proliferation, differentiation, inhibition of apoptosis, tumorigenesis and angiogenesis [21,22]. It has been demonstrated that Akt activity correlates with prostate cancer progression and poor clinical outcome [23-26]. Supporting evidence for Akt inhibition as viable prostate cancer therapy is provided by tumor growth inhibition in mice with prostate cancer [27]. In addition, it has been shown that activation of Akt also promotes androgen-independent progression of prostate cancer [28-31] and long-term androgen ablation reinforces the PI3K/Akt pathway and impedes its inhibition [32]. Therefore, suppression of the RTK/PI3K/Akt pathway is hypothesized to serve as a novel therapeutic intervention in advanced prostate cancer.

We utilized a structure-based approach to design a novel RTK inhibitor, MP470, which effectively inhibits PDGFR, c-Kit and c-Met. In contrast to Erlotinib or Imatinib, MP470 inhibits cell proliferation, induces cell growth arrest and promotes apoptosis in prostate LNCaP cancer cells. Especially when combined with Erlotinib MP470 abolished HER family/PI3K/Akt pathway with associated tumor growth inhibition in a LNCaP mouse xenograft model.

## Methods

### Cells and reagents

LNCaP, PC-3 and DU145 prostate cancer cell lines used in this study were purchased from American Type Culture Collection (Rockville, MD) and maintained in RPMI 1640 medium (Mediatech, VA) supplemented with 10% fetal bovine serum, 2 mM sodium pyruvate and 100 units/ml penicillin/streptomycin at 37°C in a humidified atmosphere containing 5% CO_2_. NIH3T3, A549 and T47D cell lines were obtained from Dr. Jesse Martinez lab (Arizona Cancer Center, University of Arizona) and maintained in the same medium as above. For the androgen-depletion experiments, LNCaP cells were grown in androgen-depleted medium, phenol red-free RPMI 1640 supplemented with 10% charcoal/dextran-treated FBS (HyClone, Logan, UT). MP470 was kindly provided by SuperGen (Dublin, CA) and Erlotinib [4-(3-ethynylphenylamino)-6,7-bis(2-methoxyethoxy)quinazolium hydrochloride] was isolated from clinical Tarceva tablets. Imatinib mesylate (IM, Gleevec) was purchased from Shanghai 21CEC Pharma. Ltd (Shanghai, China). The compounds were dissolved at 5 mM in DMSO as a stock solution, and then further diluted to desired concentrations for *in vitro *experiments. Nocodazole was purchased from Calbiochem (La Jolla, CA). Anti-PARP (H-250), anti-ErbB-3 (C-17) and anti-EGFR (1005) antibodies were obtained from Santa Cruz Biotechnology (Santa Cruz, CA). Anti-phospho-Akt (Ser473), anti-phospho-Akt (Thr308), anti-Akt, anti-phospho-p44/42 Map Kinase (Erk1/2) (Thr202/Tyr204) and anti-GAPDH (14C10) antibodies were from Cell Signaling Technology (Danvers, MA). Anti-PI-3Kinase p85 antibody was purchased from Upstate (Lake Pacific, NY). Anti-Phosphotyrosine (PY20) was from BD Biosciences (San Jose, CA). Anti-ErbB2 antibody was from Neomarkers (Fremont, CA). Anti-β-actin antibody was from Sigma (St Louis, MO).

### Analysis of cell proliferation inhibition (MTS assay)

The inhibition of cell proliferation was assessed by measuring changes in total protein in a culture of each cell line by use of a Sulforhodamine B (SRB) colorimetric assay. Briefly, cells were seeded at 8,000 for LNCaP or 4000 for PC-3 and DU145 per well onto flat-bottomed 96-well culture plates and allowed to grow for 24 hr followed by the desired treatment. After 4 days incubation, cells were quick rinsed with PBS and then fixed with 10% trichloroacetic acid (TCA) for 1 hr at 4°C. The cells were stained with 50 μl of 0.04% Sulforhodamine B (S9012, Sigma) in 1% acetic acid for 20 min at room temperature, after which the excess dye was removed by washing repeatedly with 1% acetic acid. The protein-bound dye was dissolved in 100 μl of 50 mM Tris-base solution for optical density (OD) determination at 570 nm using a microplate reader.

### Apoptosis assay

For routine analysis of apoptosis, treated cells were examined for apoptotic morphology using a fluorescence staining technique as described previously [33]. Briefly, cells were exposed to DMSO or differing doses of MP470, Erlotinib, or IM (1 to 10 μM) for 24 h and were harvested by trypsinization. After staining with a mixed dye solution containing 100 mg/ml each acridine orange and ethidium bromide the morphology of the cells was observed by fluorescence microscopy, and the number of apoptotic cells was quantified. In all cases a minimum of 200 cells were counted for each sample. Using Annexin V staining to detect apoptosis, treated cells were harvested by trypsinization and rinsed with cold PBS once. After centrifugation for 5 min, cells were resuspended in 500 μl of 1× Annexin V binding buffer (BioVision, Annexin V-FITC Reagent Kit, Cat.#1001-1000) and then added 1 μl of Annexin V-FITC and 1 μl of Propidium Iodide (BioVision, Annexin V-FITC Reagent Kit). After incubation for 5 min at room temperature in the dark, the samples were analyzed by flow cytometry.

### Cell cycle analysis

LNCaP and PC-3 cells were treated with 10 μM of Erlotinib, MP470, IM, Erlotinib plus MP470 or Erlotinib plus IM for 32 hr and then left unsynchronized or synchronized with 0.3 μg/ml Nocodazole (Sigma) for 16 hr. After treatment with trypsin-EDTA, the cells were centrifuged at 1,500 × g for 5 min at 4°C and resuspended in PBS, fixed by drop wise addition of ice-cold ethanol (100%) to a final concentration of 70%, and incubated for 30 min on ice. Fixed cells were pelleted and treated with 100 μl of RNase A (0.2 mg/ml in PBS) for 5 min at room temperature, then suspended in 1 ml ddH_2_O and boiled for 10 min in a water bath. After staining with 4 μg/ml propidium iodide, the DNA content was determined using a Becton Dickson flow cytometer and the cell cycle profile was analyzed by ModFit software. Cell aggregates were gated out of the analysis, based on the width of the propidium iodide fluorescence signal. Each profile was compiled from 10,000 gated events.

### Cell stimulation by pervanadate

Cells were cultured to 70% confluence and starved for an additional 24 hr with serum-free medium. After 4 hr pre-treatment with MP470, Erlotinib, IM or combinations at the appropriate concentrations, the cells were stimulated by pervanadate (100 μM) for 10 min and then lysed for protein analysis. Pervanadate stock solution (10 mM) was freshly prepared by adding 50 μl of 200 mM sodium orthovanadate (Na_3_VO_4_) and 250 μl of 200 mM hydrogen peroxide (diluted from a 30% stock in 20 mM HEPES, pH 7.3) to 700 μl of 20 mM HEPES (pH 7.3).

### Immunoblotting

The cells were lysed in NP-40 lysis buffer containing 50 mM Tris.Cl (pH 7.4), 0.15 M NaCl, 0.5% NP-40, 1 mM DTT, 50 mM Sodium Fluoride, and 2 μl/ml Protease inhibitor cocktail (Sigma, St. Louis, MO). Protein concentrations were determined using the BioRad protein assay kit (Hercules, CA) and 50 μg of protein was resolved by electrophoresis on a 10% SDS-PAGE gel. The proteins were then transferred onto a nitrocellulose membrane and nonspecific binding was blocked by incubating with 5% nonfat milk in TBST buffer (0.01 M Tris-Cl, 0.15 M NaCl, 0.5% Tween-20, pH 8.0) at room temperature for 1 hr. The membrane was subjected to the indicated antibodies and the proteins were detected by the SuperSignal West Pico detection system (Pierce, Rockford, IL).

### Immunoprecipitation

Cells were collected by scraping and lysed in Triton X-100 lysis buffer (50 mM HEPES, pH 7.5, 1.5 mM MgCl_2_, 150 mM NaCl, 20 mM NaF, 5 mM EDTA, 1% Triton X-100, 20 mM Na_4_P_2_O_7_, 1 mM NaVO_4_) supplemented with protease inhibitor cocktail on ice for 30 min. Lysates were clarified by centrifugation at 13,000 × g for 8 min at 4°C. Whole cell extracts were then incubated with 3 μg of PY20 anti-phosphotyrosine antibody overnight at 4°C for the immunoprecipitation experiments or resolved by SDS-PAGE and probed directly by Western blotting. Immune complexes were collected on 30 μl of protein G agarose bead (50%) slurry (Upstate) for 2 hr, washed in lysis buffer four times, and eluted by boiling in SDS sample buffer. Eluted proteins were then applied to SDS-PAGE gels and probed by Western blotting with anti-PI-3K (p85) antibody using the LI-Cor detection sysytem.

### HER2 knock-down by small interfering RNA

Neu (HER2) siRNA (sc-29405) and control siRNA (sc-37007) were purchased from Santa Cruz Biotechnology (Santa Cruz, CA). Transfection reagent (DharmaFECT3) was from Dharmacon, Inc. (Lafayette, CO). Cells were grown to 70% confluence and transfected by siRNA at a final concentration of 100 nM. 72 hr later the cells were lysed for protein analysis.

### Animal experiments

Animal care and treatment was performed at Arizona Cancer Center's experimental mouse shared services (EMSS) core facility. Forty eight 6–7 week-old SCID male mice were used. Each mouse was injected with 2× 10^7 ^LNCaP cells subcutaneously into the right hind flank. One month after inoculation, when tumors reached a volume of ~100 mm^3^, animals were divided randomly (pair-matched) into four test groups each with 12 mice: control group (DMSO), Erlotinib (80 mg/kg) group, MP470 (50 mg/kg) group and Erlotinib plus MP470 group. TKIs was administered IP daily from days 1 to 24. The control group was injected with 5% DMSO. A second study was also conducted with MP470 at 10 mg/kg and 20 mg/kg with 80 mg/kg Erlotinib to assess for biological efficacy (pharmacodynamics) and efficacy with 12 mice per group with the control arm of 5% DMSO. The length (L) and width (W) of the subcutaneous tumors were measured by calipers and the tumor volume (TV) was calculated as: TV = (L × W^2^)/2. Mice were sacrificed at the end of treatment (2–3/group), end of study or if they reached 2000 mm^3 ^at any time during the study. Excised tumors were either fixed in paraffin or snap frozen for immunohistochemical analysis.

### Immunohistochemistry

The excised tumors were fixed in 10% neutral buffered formalin (NBF) and embedded in paraffin. The 6 μM sections were deparaffinized in xylene and then rehydrated in an ethanol series to distilled water. The sections were blocked with blocking solution (0.2% BSA, 0.01% saponin and 1% normal rabbit serum in PBS) for 1 hr at room temperature. The slides were then immunostained using anti-phospho Akt (Ser473) antibody at a dilution of 1:50 in blocking solution overnight at 4°C. After washing 3 times with PBS, the secondary antibody conjugated with Cy3 (Sigma) was applied for 30 min at room temperature. The signal was checked using florescence microscopy. Primary antibody replacement with normal serum from the same animal species was used as the controls. Nuclei were stained by propidium iodide.

### Phosphorylation Antibody Array analyses

Human Phosphorylation Antibody Array (RayBiotech, Norcross, GA) was employed to assay the relative levels of phosphorylation of 71 different human RTKs after MP470 or Erlotinib or MP470 plus Erlotinib treatment. All the solutions including cell lysis buffer, blocking buffer and wash buffer were from this kit and the experiment was performed following the manufacturer's instructions. Briefly, the glass chips were blocked by 1× blocking buffer for 1 hr at room temperature and 400 μg of cell lysates were then added to the chips. After incubating at 4°C overnight, arrays were washed and incubated with biotin-conjugated anti-Phosphotyrosine for 2 hr, and then with Alexa Fluor 555-conjugated streptavidin for 2 hr. Unbound reagents were removed by washing, and the bound antibodies on the chips were visualized using the GenePix 4000B microarray scanner. The signal intensities were analyzed and relative phosphorylation levels calculated with the GenePix Pro software (Molecular Devices, Sunnyvale, CA).

### Statistical Analysis

Analysis was done using multiple t-test (with Bonferroni correction) with the STATA software package (StataCorp LP, College Station, TX). Data was analyzed by group, p = 0.05 was considered significant.

## Results

### MP470 alone or in combination with Erlotinib inhibits prostate cancer cell proliferation, promotes cell cycle arrest and apoptosis

**Figure 1:**
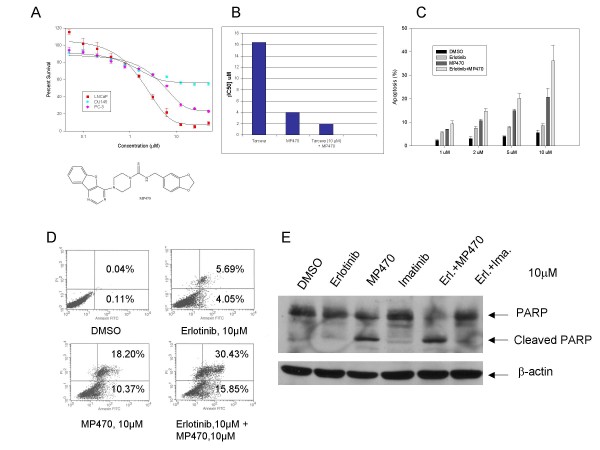
**Effects of MP470 or the MP470-Erlotinib combination on prostate cancer cell proliferation and apoptosis**. (a). LNCaP, DU145 and PC-3 cells were exposed to varying concentrations of MP470 for 4 days. Cell viability was assessed by MTS analysis. Points are the means of triplicate determinations ± SD. The IC_50 _for LNCaP and PC-3 is ~4 μM and 8 μM, respectively. Chemical structure of MP470 was shown at bottom. (b). LNCaP cells were exposed to varying concentrations of Erlotinib (Tarceva), MP470 or Erlotinib (10 μM) plus MP470 (varied concentrations) for 4 days. Cell viability was assessed by MTS analysis. IC_50 _was calculated with CalcuSyn software and the graph was shown. (c). LNCaP cells were treated with the indicated doses of Erlotinib or MP470 alone or in combination for 48 hr, and apoptosis was detected as morphologic change by fluorescent microscopy. Values are the means of three independent experiments ± SD. Control cells were treated with DMSO vehicle. (d). LNCaP cells were treated with DMSO (control), 10 μM of Erlotinib or MP470 or in combination for 48 h. Flow cytometric analysis of apoptotic fraction based on propidium iodide (Y-axis) and annexin V staining (X-axis) showed up to 28% and 46% of apoptosis was induced by MP470 alone and in combination with Erlotinib, respectively. (e). LNCaP cells were treated with the indicated doses of Erlotinib or MP470 or IM alone or the combinations for 48 hr, and PARP cleavage was determined by immunoblotting with anti-PARP antibody. β-actin was used as a loading control. MP470 or MP470-Erlotinib but not Erlotinib or IM or Erlotinib-IM combination was shown to cause PARP cleavage in LNCaP cells.

MP470, a novel receptor tyrosine kinase (RTK) inhibitor has shown growth inhibitory activity against a variety of cancer cell lines. MP470 is currently in Phase I clinical trial testing. In this study, the cytotoxicity of MP470 was evaluated on prostate cancer cell lines (LNCaP, PC-3 and DU-145). The drug was effective on LNCaP and PC-3 cells with an IC_50 _of ~4 μM and 8 μM, respectively. However, MP470 had only a modest effect on the viability of DU-145 cells (Fig. 1A). Here we focused on LNCaP cells as it is the most widely used *in vitro *model of prostate cancer [34]. Since growing evidence implicates the HER family in prostate cancer progression, we evaluated the cytotoxic effect of Erlotinib [EGFR/HER1 tyrosine kinase inhibitor (TKI)] on LNCaP cells and demonstrated a cytotoxic effect with an IC_50 _of >10 μM. However, when Erlotinib (10 μM) was combined with varying doses of MP470, the IC_50 _of MP470 decreased to 2 μM (Fig. 1B). This indicates that Erlotinib has an additive effect on the cytotoxicity of MP470.

We next examined whether apoptosis is involved in the inhibition of cell proliferation by MP470. LNCaP cells were treated with DMSO and increasing doses of MP470 (1, 2, 5, 10 μM) alone or in combination with Erlotinib for 48 hr. Apoptosis quantified by morphologic changes was induced in a dose-dependent manner and this effect was synergistic with Erlotinib (Fig. 1C). Treatment of LNCaP cells with either Erlotinib (10 μM) or MP470 (10 μM) induced 9% or 21% apoptosis respectively, while apoptosis with the combination, increased to 36%. These morphologic changes were confirmed by Annexin V staining and PARP cleavage assays (Fig. 1D and 1E) respectively. Because MP470 inhibits c-Kit and PDGFR RTKs, we evaluated Imatinib Mesylate (IM), a well-established c-Kit and PDGFR TKI. IM had an IC_50 _of ~12 μM in LNCaP cells [35] similar to that observed for Erlotinib alone. Interestingly, IM did not induce apoptosis in LNCaP cells either alone or in combination with Erlotinib (Fig. 1E). This implies that c-Kit and PDGFR do not play a role in protecting apoptosis and that MP470 inhibits LNCaP cells by a mechanism independent of c-Kit and PDGFR.

**Figure 2:**
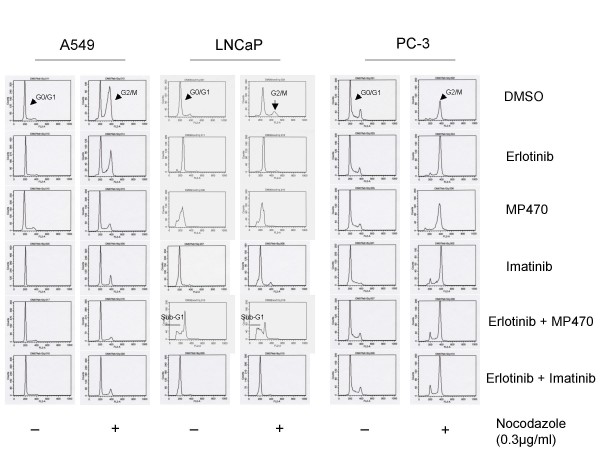
**Cell cycle arrest induced by MP470. A549, LNCaP and PC-3 cells were treated with DMSO, 10 μM of Erlotinib, or MP470, or IM alone or the combinations for 32 hr and then treated with or without nocodazole for an additional 16 hr**. Cells were harvested, fixed, treated with RNase, and then labeled with propidium iodide, and analyzed by flow cytometry (X-axis: DNA content, Y-axis: cell numbers). MP470 induced a G_1 _arrest in A549 and LNCaP but not in PC-3 cells.

In order to glean whether MP470 inhibits cell cycle progression, we treated the lung cancer cell line A549 and two prostate cell lines, LNCaP and PC-3 with DMSO, 10 μM of Erlotinib, MP470, IM or combinations for 32 hr. The cells were then left unsynchronized or synchronized at the mitotic phase by nocodazole for 16 hr. Cell cycle progression analyzed by flow cytometry (Fig. 2) showed that MP470 induced G_1 _arrest in A549 and LNCaP cells as they cannot be synchronized in G_2_/M by nocodazole compared to DMSO control. However, MP470 did not induce G_1 _arrest in PC-3 cells, implicating that this arrest is cell line specific. In addition, consistent with the above apoptosis data, we also observed a sub-G_1 _population in cells treated with Erlotinib plus MP470 (Fig. 2). Together, our data indicate that MP470 has inhibitory effects on cell growth and cell cycle progression, promotes apoptosis and that these effects are enhanced by Erlotinib.

### A combination of MP470 and Erlotinib causes a further decrease in Akt activity compared with MP470 alone

**Figure 3:**
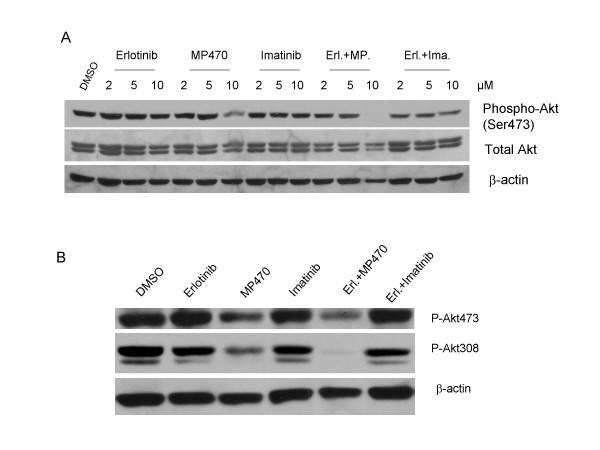
**Effects of MP470, Erlotinib, and MP470-Erlotinib treatment on Akt activity**. (a). LNCaP cells were treated with DMSO or different doses of Erlotinib, MP470, IM or combinations as indicated for 30 hr. Phospho(Ser473)-Akt and total Akt were detected by immunoblotting. β-actin antibody was used as the loading control. (b). LNCaP cells were grown in androgen-depleted medium, phenol red-free RPMI 1640 supplemented with 10% charcoal/dextran-treated FBS for 10 days. The cells were treated with 10 μM of Erlotinib, MP470, IM alone or Erlotinib plus MP470 and Erlotinib plus IM for 24 hr, and Akt phosphorylation was analyzed by Western blotting.

Since MP470 or MP470 plus Erlotinib inhibited LNCaP cell survival, we evaluated whether MP470 or MP470 plus Erlotinib could inhibit Akt activation. As shown in figure 3A, Akt activity (as measured by phosphorylation on Ser473) was significantly reduced by 10 μM MP470 alone but was not reduced by Erlotinib or IM. Moreover, MP470 plus Erlotinib completely abolished Akt phosphorylation in LNCaP cells with an unchanged total protein level of Akt. It has been reported that PI3K and Akt activities are increased following androgen deprivation [32], and activation of this pathway plays an essential role in the androgen-refractory progression of prostate cancer by enhanced cell proliferation and survival [30]. To further determine whether MP470 or combination with Erlotinib continues to inhibit Akt activity after androgen deprivation, LNCaP cells were cultured in androgen-free medium for 10 days and then treated with MP470, IM and Erlotinib alone or in combination. Consistent with previous studies, the phosphorylation of Akt at both Ser473 and Thr308 was increased dramatically after androgen deprivation (Fig. 3B). MP470, especially in combination with Erlotinib continues to inhibit these activating phosphorylation events following androgen deprivation. However, Erlotinib or IM alone or combination had no effect on Akt phosphorylation.

### MP470 plus Erlotinib Inhibits HER1, 2 and 3 phosphorylation

**Figure 4:**
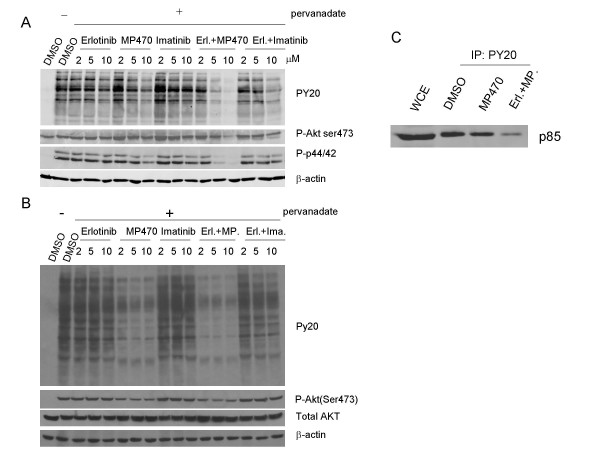
**MP470-Erlotinib combination markedly reduces protein kinase activity**. (a-b). LNCaP (a) and NIH3T3 (b) cells were serum starved for 24 hr, pretreated with drugs as indicated for 2 hr, and then treated with pervanadate (100 μM) for 10 min. Whole cell extracts were analyzed by immunoblotting for phosphorylated tyrosine kinases, phosphorylated Akt (Ser473), phosphorylated ERK1/2 (Thr202/Tyr204), and total Akt. (c). LNCaP cells were serum-starved for 24 hr, pretreated with DMSO, 10 μM of MP470 or MP470-Erlotinib, and then stimulated by pervanadate for 10 min. For immunoprecipitation (IP) assays, whole cell extracts (WCE) containing equal amounts of protein were incubated with anti-phosphotyrosine (PY20) antibodies overnight at 4°C. Immune complexes were enriched by Protein G-Agarose beads and probed by Western blotting for the p85 subunit of PI3K.

Because MP470 or the combination of MP470 and Erlotinib inhibits Akt phosphorylation, we next addressed whether they affect the upstream components of the Akt pathway. LNCaP and NIH3T3 cells were serum-starved for 24 hr, pre-treated with Erlotinib or MP470 or IM, Erlotinib plus MP470 or Erlotinib plus IM at 2, 5 and 10 μM for 4 hr, and then treated for 10 min with 100 μM pervanadate, a global protein tyrosine phosphatase inhibitor that is commonly used to maintain tyrosine kinase phosphorylation in cells [36]. Initially, we detected the total phosphotyrosine level by anti-phosphotyrosine antibody (PY20) which showed a dramatic increase in phosphorylation after pervanadate treatment (Fig. 4A and 4B). MP470 alone or MP470 plus Erlotinib decreased total tyrosine phosphorylation (Fig. 4A and 4B). Concomitantly, Akt and Erk phosphorylation were also reduced by MP470 or MP470 plus Erlotinib (Fig. 4A, B). Further, MP470 plus Erlotinib blocked the interaction between the PI3K p85 subunit and phosphorylated tyrosine kinases, an essential process for PI3K activation (Fig. 4C). In contrast, Erlotinib and IM had no effect on tyrosine or Akt phosphorylation, even when combined.

**Figure 5:**
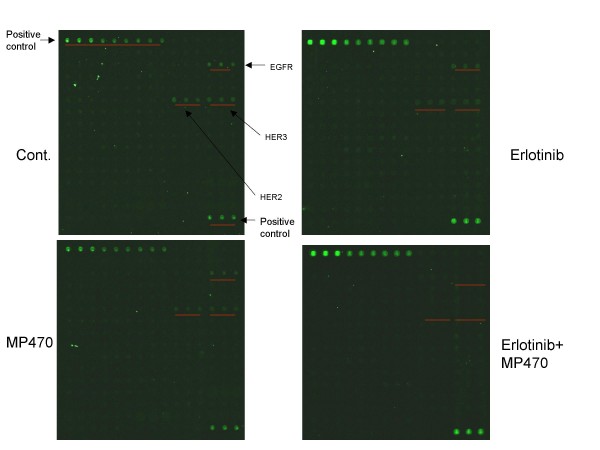
**Effects of MP470-Erlotinib combination on phosphorylation of different human RTKs**. LNCaP cells were serum starved for 24 hr, pretreated with DMSO (control), Erlotinib (10 μM), MP470 (10 μM), or MP470-Erlotinib (10 μM each) for 2 hr, and then treated with pervanadate (100 μM) for 10 min. For Phosphorylation Antibody Array analysis, 400 μg of cell lysates were incubated with the glass chips at 4°C overnight. After washing for several times, the arrays were incubated with biotin-conjugated anti-Phosphotyrosine for 2 hr, and then with Alexa Fluor 555-conjugated streptavidin for 2 hr. Unbound reagents were removed by washing, and the bound antibodies on the chips were visualized using the GenePix 4000B microarray scanner.

**Figure 6:**
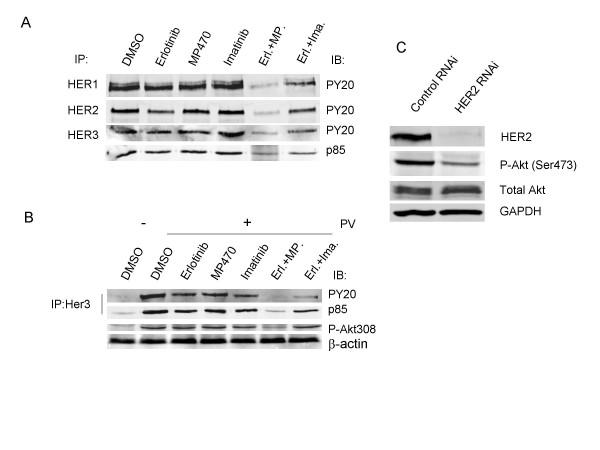
**Effects of MP470-Erlotinib combination on tyrosine phosphorylation of the HER family and PI3K/Akt**. LNCaP (a) and T47D (b) cells were serum starved for 24 hr, pretreated with 10 μM drug as indicated for 2 hr, and then treated with pervanadate (100 μM) for 10 min. Cell extracts were incubated with anti-EGFR, anti-HER2 and anti-HER3 antibodies at 4°C overnight. The immune complexes were enriched by Protein G-Agarose beads and probed by immunoblotting for phosphotyrosine (PY20) and the p85 regularly subunit of PI3K. Western blotting analysis for phosphorylated Akt was performed in T47D cells. (c). SiRNA knockdown of HER2 decreased phosphorylated Akt. LNCaP cells were grown to 70% confluence and treated with non-targeting siRNA (control RNAi) and siRNA against HER2 at a concentration of 100 nmol/L. At 72 hr, cells were harvested to detect HER2, phosphorylated Akt and total Akt by Western blotting. GAPDH was used as a loading control.

Since RTKs bind and activate PI3K and then Akt, we further attempted to identify the RTKs which were targeted by MP470 or MP470 plus Erlotinib. A phosphorylation antibody array specifically designed to simultaneously identify the relative levels of phosphorylation of 71 different human RTKs was performed. Interestingly, the HER family of receptors including the HER1 (EGFR), HER2 and HER3 was found to be affected (Fig. 5). To confirm these, co-immunoprecipitation and immunoblotting were performed and the results showed that phosphorylation of HER1, 2 and 3, binding of HER3 to PI3K p85, as well as downstream Akt activity were dramatically suppressed by MP470 plus Erlotinib in LNCaP (Fig. 6A) and T47D breast cancer cells (Fig. 6B). To further study whether HER family inhibition is involved in the regulation of Akt phosphorylation, we utilized small interference RNA (siRNA) to knockdown HER2 in LNCaP cells which is highly expressed compared to HER1 and HER3, and the data showed that Akt phosphorylation was decreased after HER2 knockdown (Fig. 6C). Together, these data imply that MP470 plus Erlotinib exquisitely inhibits cell survival through the HER family/PI3K/Akt pathway.

### MP470 plus Erlotinib inhibits prostate cancer tumor growth in xenograft mice

**Figure 7:**
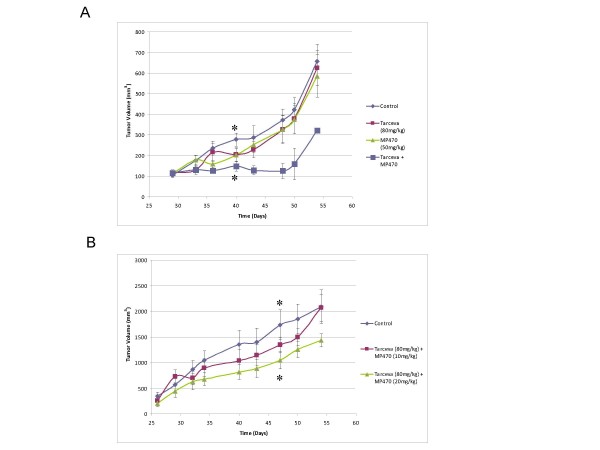
**The MP470-Erlotinib combination causes TGI in an LNCaP xenograft mouse model**. (a). 2 × 10^7 ^LNCaP cells were injected into the flanks of SCID male mice and tumors grown for 4 weeks to achieve a volume of ~100 mm^3^. The mice were pair matched into different groups (12 mice/group). The mice were treated with DMSO, Erlotinib (80 mg/kg), MP470 (50 mg/kg), Erlotinib (80 mg/kg) plus MP470 (50 mg/kg) IP daily for 14 days (starting day 30 and ending day 44), and mean tumor volume ± SEM are graphed. * *P*-value = 0.008. (b). 2 × 10^7 ^LNCaP cells were injected into the flanks of SCID male mice and three groups (12 mice/group) were pair matched when the tumors grew to ~300 mm^3^. The mice were treated with DMSO (control), Erlotinib (80 mg/kg) plus MP470 (10 mg/kg or 20 mg/kg, respectively) by IP daily for 22 days (starting day 27 and ending day 49), and the mean tumor volume ± SEM are graphed. * *P*-value = 0.01.

**Figure 8:**
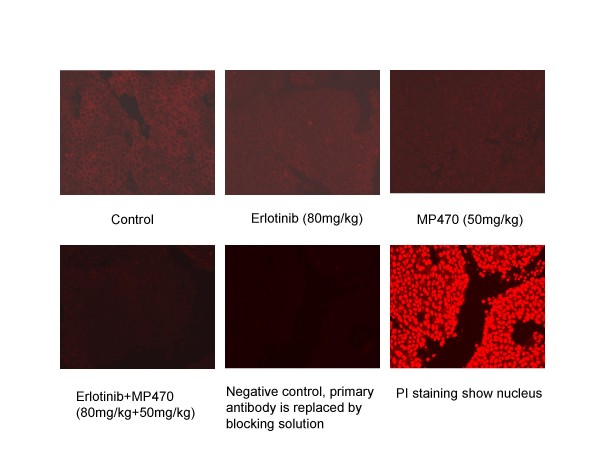
**Effects of MP470-Erlotinib combination on Akt phosphorylation in xenograft tissues**. IHC analysis for Akt activity was performed on the tumors harvested at the end of the treatment of an LNCaP xenograft mouse model. Paraffin embedded sections were immuno-stained for expression of phosphorylated Akt (Ser473). Tumors from mice treated with the MP470-Erlotinib combination had a near complete inhibition of Akt phosphorylation compared to control or individual therapies (200 ×).

We then evaluated the safety and efficacy of MP470, Erlotinib and MP470 plus Erlotinib in a mouse LNCaP xenograft model based on the cell culture mechanism of action studies. Four LNCaP xenograft arms each with 12 mice were dosed intraperitoneally with DMSO (control) or Erlotinib 80 mg/kg or MP470 50 mg/kg or Erlotinib 80 mg/kg plus MP470 50 mg/kg daily for 2 weeks and then observed for a further 11 days (Fig. 7A). Individual therapy with MP470 or Erlotinib showed modest tumor growth inhibition (TGI), while MP470 plus Erlotinib had a marked effect on TGI (45–65%). However, due to the high doses of MP470 used, only five or one mouse remained alive in the combination arm at the end of treatment or at the end of the study, respectively. We therefore reduced the MP470 dose to 10 mg/kg or 20 mg/kg for the combination treatment. As shown in figure 7B, TGI in the group receiving 10 mg/kg MP470 + 80 mg/kg Erlotinib was not significantly different from the control group. However, mice receiving 20 mg/kg MP470 + 80 mg/kg Erlotinib had a significant TGI compared to the control group (p = 0.01). To determine whether the biological effect(s) of MP470 plus Erlotinib are correlated to its ability to inhibit Akt activation, Akt phosphorylation in tumor tissue at the end of treatment from the different treatment groups was analyzed by immunohistochemistry. Figure 8 showed Akt phosphorylation was abolished in the combination arm (MP470 plus Erlotinib) compared to control or individual therapies. Together, these observations indicate that the combination of MP470 and Erlotinib inhibits Akt with an associated TGI.

## Discussion

Single agent therapy with small molecule TKIs is effective in malignancies dependent on mutated constitutively activated RTKs [e.g. Kit/a-PDGFR in GIST (IM, Sunitinib), EGFR in NSCLC and GBM (Erlotinib)] and non-RTKs such as, Bcr-Abl in CML (IM, Dasatinib). However, chronic therapy with a single TKI eventually becomes ineffective due to acquired mechanisms of resistance. In contrast, single agent TKIs is less effective in tumors that amplify and over-express RTKs such as the EGFR family (HER1 in head and neck cancer, HER2 in breast cancer, HER1 and 2 in pancreas cancer). Clinical efficacy studies reported that the HER1 selective Erlotinib and Gefitinib, the HER1/HER2 selective Lapatanib and the pan-HER selective Canertinib have shown limited activity in the treatment of HER2 over-expressing breast cancer, despite evidence suggesting these cancers are highly dependent on HER2 function [37]. Correlative data from tumor biopsies confirm that TKIs reach their molecular targets and suppress the activity of EGFR, HER2 and MAPK signaling. However, inactivation of Akt signaling is not apparent suggesting that HER2 signaling is not completely suppressed by these therapies. Therefore, critical studies are required to determine mechanisms by which the HER family over expressing tumors evade targeted therapy and to identify novel combination TKI therapies to suppress the PI3K/AKT survival pathway. In this study, cell-based evaluation showed that MP470, a novel tyrosine kinase inhibitor inhibited cell proliferation, induced growth arrest and promoted apoptosis in prostate cancer cells. In addition, the combination treatment of MP470 and Erlotinib completely inhibited HER family activation, and the downstream signaling pathway PI3K/Akt in LNCaP and T47D cells. Moreover, MP470 plus Erlotinib significantly suppressed tumor growth in an LNCaP mouse xenograft model, suggesting it could be used as a new combination for prostate cancer treatment.

In prostate cancer, Akt has been shown to be constitutively activated due to loss of PTEN, which negatively regulates PI3K. Clinical reports indicate that Akt is significantly over-expressed in prostate tumors compared to benign prostatic tissue, and its level is directly correlated with tumor progression and prostate-specific antigen (PSA) serum levels, as well as a higher Gleason score [23,26]. In addition, increased phosphorylation of Akt (Ser473) has been shown to be an excellent predictor of poor clinical outcome in prostate cancer [25]. Moreover, stable over-expression of constitutively active Akt dramatically enhances LNCaP xenograft tumor growth in intact male nude mice [24]. In contrast, inhibition of PI3K or Akt induces apoptosis in LNCaP cells and tumor growth suppression *in vivo *[38,39]. Consequently, Akt inhibition is a rational therapy or an endpoint of therapy in prostate cancer. Indeed, clinical studies with agents known to act through Akt inhibition show promise [40]. Consistent with these, in this study we showed that an MP470-Erlotinib combination completely inhibits Akt activity which may contribute to the tumor suppression seen in an LNCaP xenograft mouse model. In addition, hormone-refractory prostate cancer is a major clinical obstacle as there are no drugs to halt its progression [3]. Previous studies have shown that PI3K/Akt activation is associated with prostate cancer progression from an androgen-dependent to an androgen-independent state [30]. In androgen-ablated LNCaP cells, PI3K/Akt activity is elevated and required for growth and survival [31] and inhibition can restore sensitivity to apoptosis induction [32]. In a mouse xenograft model of LNCaP, conditional Akt activation promotes tumor growth in castrated animals by enhanced cell proliferation and inhibition of apoptosis [28]. Thus, blockage of Akt activity should prove beneficial for hormone-refractory prostate cancer. Our experiments showed that the MP470-Erlotinib combination efficiently inhibited Akt activity in androgen-ablated LNCaP cells (Fig. 3B), suggesting that this combination may be a viable treatment modality in patients failing androgen blockade or can be administered with androgens in front-line therapy to prevent hormone refractory status.

Except for the loss of PTEN function, PI3K/Akt signaling is often dysregulated in human cancer due to constitutive activation of receptor tyrosine kinases (RTKs) [41]. Of the known RTKs, activation of the HER family (HER1, 2 and 3) and the PDGFR family has been demonstrated to associate with prostate cancer progression [16,19]. In prostate cancer cell lines, HER family receptors are over-expressed [42,43] and inhibition with specific TKIs has shown anti-tumor effects *in vitro *and *in vivo *[44,45]. HER family members are also widely expressed in cancerous tissues of the prostate and significant over-expression is found in hormone-refractory prostate cancer and metastatic tissue compared to localized prostate cancer [46]. Hence, HER family receptors have become potential therapeutic targets in prostate cancer [47]. MP470, designed as an ATP-competitive TKI was very effective in inhibiting tyrosine phosphorylation in LNCaP and NIH3T3 cells after pervanadate stimulation (Fig. 4A and 4B). Further, the MP470-Erlotinib combination completely inhibited tyrosine phosphorylation and p85 binding (Fig. 4C) as well as Akt activity. The RTK phospho-antibody assay identified the HER family (HER1, HER2 and HER3) in LNCaP cells as targeted by MP470 (Fig. 5A). Erlotinib or MP470 alone did not totally inhibit phosphorylation of the HER family. However, MP470-Erlotinib combination completely inhibited the phosphorylation of HER1, HER2 and HER3, the binding of PI3K regulatory subunit p85 to HER3 and downstream Akt activity (Fig. 5B and 5C). Due to the cross-talk between the individual members of the HER family or between the HER family and other RTKs, evidence indicates that targeting a single RTK is inadequate as a therapeutic modality in cancer therapy [48,49]. In gefitinib-resistant NSCLC cell lines, c-Met, an oncogenic RTK phosphorylates HER3 and leads to activation of the PI3K/Akt pathway. Treatment of the resistant cells with a TKI specific for c-Met or gefitinib alone did not inhibit cell viability or affect HER3 and Akt phosphorylation. However, the combination of both drugs inhibited resistant cell growth and prevented HER3 and Akt phosphorylation [50]. Because MP470 does inhibit c-Met activation (data not shown), as well as c-Kit and Axl[51], it is likely that one or more of these RTKs cross-talk with the HER family members and activate them. Therefore, inhibition of HER1 and HER2 by Erlotinib and multi-targeted RTK inhibition by MP470 may explain the complete inhibition of the HER3/PI3K/Akt pathway by Erlotinib-MP470 combination in LNCaP cells. However, further studies are required to identify potential target(s) of MP470 in LNCaP cells for confirming this hypothesis.

## Conclusion

MP470, a novel receptor tyrosine kinase inhibitor effectively inhibits cell proliferation in prostate cancer cell lines. When combined with Erlotinib, MP470 induced apoptosis and cell growth arrest with abolition of tumor growth in a dose-dependent manner in an LNCaP xenograft mouse model. The HER family and the phosphorylation of downstream Akt are inhibited by this novel TKI combination. Hence, blockade of HER family/PI3K/Akt may represent a useful treatment modality for prostate cancer. The safety and efficacy of the MP470-Erlotinib combination is currently being evaluated in a Phase I clinical trial for refractory solid tumors and results are awaited with enthusiasm.

## Competing interests

The authors except Dr. David Bearss declare that they have no competing interests. Dr. David Bearss has stocks and shares and is the Chief Scientific Officer at SuperGen.

## Authors' contributions

WQ and DM were responsible for designing the experiments, analyzing the data and drafting the manuscript. WQ also performed the apoptosis assay, immunoblotting, immunoprecipitation, immunohistochemistry, knock-down of HER2, cell cycle and phosphorylation antibody array analyses. LC, AS and KDC carried out MTS analyses. CJR did statistical analysis. JWS and DB gave advice on the experiments and manuscript. All authors read and approved the final manuscript.

## Pre-publication history

The pre-publication history for this paper can be accessed here:

http://www.biomedcentral.com/1471-2407/9/142/prepub
